# Odor-active volatile compounds in preterm breastmilk

**DOI:** 10.1038/s41390-021-01556-w

**Published:** 2021-05-07

**Authors:** Mariana Muelbert, Laura Galante, Tanith Alexander, Jane E. Harding, Chris Pook, Frank H. Bloomfield

**Affiliations:** 1 Liggins Institute, University of Auckland, Auckland, New Zealand; 2 Neonatal Unit, Kidz First, Middlemore Hospital, Auckland, New Zealand

## Abstract

**Background:**

Volatile compounds in breastmilk (BM) likely influence flavor learning and, through the cephalic phase response, metabolism, and digestion. Little is known about the volatile compounds present in preterm BM. We investigated whether maternal or infant characteristics are associated with the profile of volatile compounds in preterm BM.

**Methods:**

Using solid-phase microextraction coupled with gas chromatography/mass spectrometry, we analyzed volatile compounds in 400 BM samples collected from 170 mothers of preterm infants.

**Results:**

Forty volatile compounds were detected, mostly fatty acids and their esters (FA and FAe), volatile organic compounds (VOCs), aldehydes, terpenoids, alcohols, and ketones. The relative concentration of most FA and FAe increased with advancing lactation and were lower in BM of most socially deprived mothers and those with gestational diabetes (*p* < 0.05), but medium-chain FAs were higher in colostrum compared to transitional BM (*p* < 0.001). Infant sex, gestational age, and size at birth were not associated with the profile of volatile compounds in preterm BM.

**Conclusions:**

Sensory-active volatile FA and FAe are the major contributors to the smell of preterm BM. The associations between lactation stage, maternal characteristics, and volatile compounds, and whether differences in volatile compounds may affect feeding behavior or metabolism, requires further research.

**Impact:**

- Sensory-active volatile FAs are major contributors to the smell of preterm BM and are influenced by the lactation stage and maternal characteristics.
- Longitudinal analysis of volatile compounds in preterm BM found that FAs increased with advancing lactation.
- Colostrum had a higher concentration of medium-chain FAs compared to transitional BM and the concentration of these is associated with socioeconomic status, gestational diabetes, and ethnicity.

## Introduction

Breastmilk (BM) is considered the gold standard in neonatal nutrition^1^ and breastfeeding provides several nutritional and health benefits to infants and mothers, leading to the general consensus that BM has many advantages over other feeding methods.^2,3^

Volatile compounds originating from maternal diet and metabolism can contribute to the flavor and composition of amniotic fluid and BM,^4–6^ influencing fetal and infant flavor learning and later infant feeding preferences.^7,8^ For example, infants have been reported to demonstrate greater acceptance of weaning foods that mothers consumed during late gestation and lactation.^4,9^ Neonates are capable of recognizing odors from their own amniotic fluid and from their own mother’s milk^6,10^ and can distinguish between the smell of BM and infant formula, demonstrated by increased sucking behavior^11,12^ and cerebral oxygenation in response to BM.^13^ Hence, BM may be a link between the antenatal and postnatal sensory environments.^8^

The smell and taste of food are intimately related to appreciation of food and digestion. Sensory cues from food assist digestion and metabolism by triggering a cascade of physiological responses that lead to increased salivation and peristaltic movements and the release of hormones and enzymes related to digestion.^14^ Promotion of these physiological responses has the potential to enhance tolerance and metabolism of enteral feeds in preterm infants in whom establishment of milk feeds can be a major determinant of hospital length of stay. There is some evidence that exposure to smell and taste of milk may reduce duration of hospital stay in preterm infants,^15^ but the quality of evidence is low.

The composition of BM is highly variable and can be affected by lactation stage,^16,17^ stage of a feed (fore- versus hind-milk),^18^ circadian rhythm,^19,20^ maternal diet, nutritional supplementation and medical conditions,^17,21,22^ maternal anthropometry, parity, socioeconomic status,^23,24^ and infant sex,^17,25^ although the strength of the evidence for maternal and infant factors is less strong. Length of gestation also influences the composition of BM^16^ with mothers of preterm infants producing BM in the first 12–16 postnatal weeks with higher energy and protein content,^16^ lower lactose,^26^ and a different profile of fatty acids (FA), especially medium-chain FAs (MCFAs),^27^ compared to BM produced by mothers of full-term (FT) infants. Environmental contaminants and toxins (e.g., alcohol and recreational drugs) also have been detected in BM and have the potential to impact upon infant growth and development.^28,29^ However, there is much less evidence around how the volatile compounds present in BM responsible for smell, taste, and activation of the cephalic phase response vary according to different maternal, pregnancy, and infant factors.

We recently demonstrated that the profile of volatile compounds in different milk types commonly fed to preterm infants (BM, fortified BM, pasteurized donor BM, and a variety of infant formulas) are markedly distinct.^30^ However, that study only analyzed a small number of samples from preterm BM, all collected in the first two weeks postpartum, as the main purpose was to compare different types of milk. Therefore, this study aims to investigate maternal and infant factors that may be associated with the profile of volatile compounds in BM produced by mothers of moderate- and late-preterm (MLP) infants enrolled in a multicenter randomized controlled trial, the DIAMOND trial. We hypothesized that the profile of volatile compounds would be influenced by lactation stage, length of gestation, infant sex, birth weight, and maternal characteristics such as age, ethnicity, socioeconomic status, and medical condition.

## Methods

### Study population

This is a cohort study nested within the DIAMOND trial (ACTRN12616001199404),^31^ a multicenter, factorial, randomized controlled trial investigating the impact of different nutritional approaches on feed tolerance, body composition, and neurodevelopment in MLP infants. The randomization factors are (1) provision of intravenous parenteral nutrition compared to intravenous dextrose alone; (2) provision of enteral nutrition with exclusive maternal BM compared to milk supplementation (with infant formula or donor BM), and (3) exposure to smell and taste of milk before all gastric tube feeds compared to standard care (no exposure to smell and taste of milk prior to tube feeds). Eligible infants were born between 32^+0^ and 35^+6^ weeks’ gestation, admitted to one of five neonatal care units (NCUs) in New Zealand, had intravenous access secured for clinical reasons, and whose mothers intended to breastfeed. Infants with a congenital abnormality or for whom a particular mode of nutrition was clinically indicated were not eligible. This research was approved by the National Health and Disability Ethics Committee (HDEC 16/NTA/90) and written informed consent was obtained from parents or caregivers.

Maternal ethnicity, education, postcode, and clinical information (gestational diabetes, antenatal steroid administration, delivery mode), and infant characteristics (sex, gestational age at birth, anthropometric measures) were collected prospectively. In New Zealand, postcode of domicile is used to generate a social deprivation index (the New Zealand Deprivation (NZdep) Index) in the Classification Coding System from Statistics New Zealand^32^ with a scale from 1 to 10, representing low to high social deprivation. NZdep index data were divided into quintiles, the first (Q1) and fifth quintiles (Q5) representing least and most socially deprived areas, respectively. Ethnicity was self-identified and prioritized according to New Zealand’s Ministry of Health protocols.^33^ For analysis, ethnicity was grouped into Caucasian/European; Asian (Asia, South East Asia, and Indian subcontinent); Pasifika (South Western Pacific); Māori (New Zealand Māori); and Other (ethnicities not defined in the previously mentioned categories).

Gestational diabetes mellitus (GDM) was classified according to the New Zealand Ministry of Health criteria of fasting glucose ≥5.5 mmol/L (≥99 mg/dL) or glucose ≥9.0 mmol/L (≥162 mg/dL) 2 h following a 75 g oral glucose tolerance test. Antenatal steroid course was classified as complete (>1 dose given, with the first dose >24 h before birth), incomplete (1 dose given <24 h before birth), or no antenatal steroid received.

### Sample collection and preparation

Mothers of infants enrolled in the trial were asked to provide a sample of BM at postnatal days 3 (+2), 5 (+2), and 10 (±2), and at follow-up visit at 4 months (±2 weeks) corrected age (CA, counting from full term at 40 weeks)). The lactation stage was defined as colostrum (samples collected at postnatal days 3 and 5), transitional BM (samples collected at postnatal day 10), and mature BM (samples collected at 4 months’ CA). The cohort reported here includes mothers who provided at least one BM sample during the study period and their infants. More details on BM sample collection can be found elsewhere.^34^ Briefly, mothers were asked to express BM from their right breast until completely empty, using a hospital-grade electronic breast pump (Medela, Baar, Switzerland). Sample collection occurred in the morning at least 2–3 h after the previous BM expression or breastfeeding. After the right breast was completely emptied, the total volume of expressed BM was vortexed for 2 min at high speed to ensure homogeneity and 2 mL were collected using a sterile syringe (brands varied according to collection site). Equal amounts of BM were aliquoted into four low protein binding microtubes (Eppendorf, Germany), one aliquot of which was used in this study. Samples were frozen immediately after collection and stored at −20 °C for up to 2 weeks in a dedicated freezer in each NCU, then transferred to the Liggins Institute, and stored at −80 °C freezer for 12–24 months until analysis.

Samples were randomly allocated into batches for analysis. Aliquots of BM were thawed at 4 °C for 2 h and vortexed at low speed to ensure homogeneity before 400 μL was transferred into 10 mL headspace amber vials with magnetic screw caps and polytetrafluoroethylene-lined silicone septa (Thermo Fisher Scientific Inc., New Zealand). As limited preterm BM was available, pasteurized BM (PBM) from a single deidentified donor was used as quality control (QC) and processed in the same way as the study samples. PBM was obtained from Christchurch Milk Bank, New Zealand, shipped frozen, and then stored at −20 °C until analysis. More information about the Human Milk Bank protocols can be found elsewhere.^35^ Procedural blanks consisted of 400 μL ultrapure water (MilliQ). The procedural blanks and QCs were placed at the beginning, middle, and end of each batch. All samples, QCs, and blanks were spiked with 10 μL of ultrapure water containing 2-chlorophenol at 20 μL/L as an internal standard (IS) (Sigma-Aldrich, Sydney, Australia). Data from the QC analyses were processed in parallel with the samples to confirm the reproducibility of retention times and peak areas among batches.

### Analysis of volatile compounds

Untargeted analysis of volatile compounds was performed by headspace solid-phase microextraction gas chromatography with mass spectrometry (SPME-GC-MS) using a Thermo Fisher Trace GC Ultra with a Programmable Temperature Vaporizer connected to a Thermo ISQ mass spectrometer as described previously.^30^ In brief, a Gerstel MPS2 autosampler was used to equilibrate samples at 37 °C, with continuous agitation, for 10 min. The SPME fiber was divinylbenzene-carboxen-polydimethylsiloxane 50/30 μm (Supelco, Bellefonte, Pennsylvania) measuring 20 mm in length. A 10 mL amber vial was used to prevent contact between the milk and the SPME fiber. Extraction of volatile compounds occurred for 10 min. The carrier gas was zero-grade helium (99.995%, BOC New Zealand) at a constant flow rate of 1.1 mL/min. Upon injection, the SPME fiber was simultaneously desorbed and conditioned in the GC injector in high-pressure splitless mode using a low-volume SPME-specific deactivated liner (0.75 mm ID) at 250 °C for 10 min. The column was a Phenomenex 1701 capillary column (30 m × 250 μm × 0.25 μm). The oven temperature was set at an initial temperature of 35 °C and held for 4 min, followed by an increase of 5 °C/min up to 165 °C, followed by an increase of 50 °C/min up to 265 °C. Data were acquired at a scan rate of 5 Hz in the range *m*/*z* 20–300.

Deconvolution, integration, and identification of features in the GC-MS data were performed using Agilent MassHunter Unknowns software version B10.00 (Agilent Technologies, Santa Clara, California) searching the 2020 version of the NIST mass spectral library (National Institute of Standards & Technology, Gaithersburg, Maryland) using retention time calibration by Kovats Index and a match factor threshold of 80%. Identities were filtered to exclude features with a signal-to-noise ratio <10 or that were identified in <1% of samples. Authentic standards were run to verify the identities of short-chain FA (SCFAs) and MCFAs and their esters (FAe), as well as various alcohols, aldehydes, and ketones for which standards were readily available. These annotations comply with the criteria for level 1 metabolite identification described by the Metabolomics Standards Initiative.^36^ All other annotations complied with level 2 metabolite identification. Relative quantification of compounds was performed as a function of the area under the peak for a compound in relation to the peak area of a known compound (IS). Identified peaks in each sample were blank-subtracted to remove background noise and quantified relative to the area of the IS peak in that sample (2-chlorophenol). This technique reveals differences in concentration of identified compounds between samples, expressed as relative peak area, but does not provide their absolute concentration.

### Statistical analysis

We investigated the associations between the relative concentration of volatile compounds and variables of interest (lactation stage, ethnicity, NZdep, gestational diabetes, antenatal steroid course, infant sex, and gestation at birth), using mixed model regression analysis. To account for within-subject variability and repeated measurements, participant ID and lactation stage were included as random effects. In all models, compound symmetry was assumed. A false discovery rate-adjusted *p* value of <0.05 (FDR = 0.05, Benjamini–Hochberg) was considered statistically significant for multiple comparisons. The relative concentration of identified compounds is presented as mean peak area and standard deviation. Given the exploratory nature of this study and semi-quantitative approach, no formal sample size calculation was performed a priori. Statistical analysis was conducted using R programming environment version 3.6.1.^37^

## Results

### Study population

**Fig. 1:**
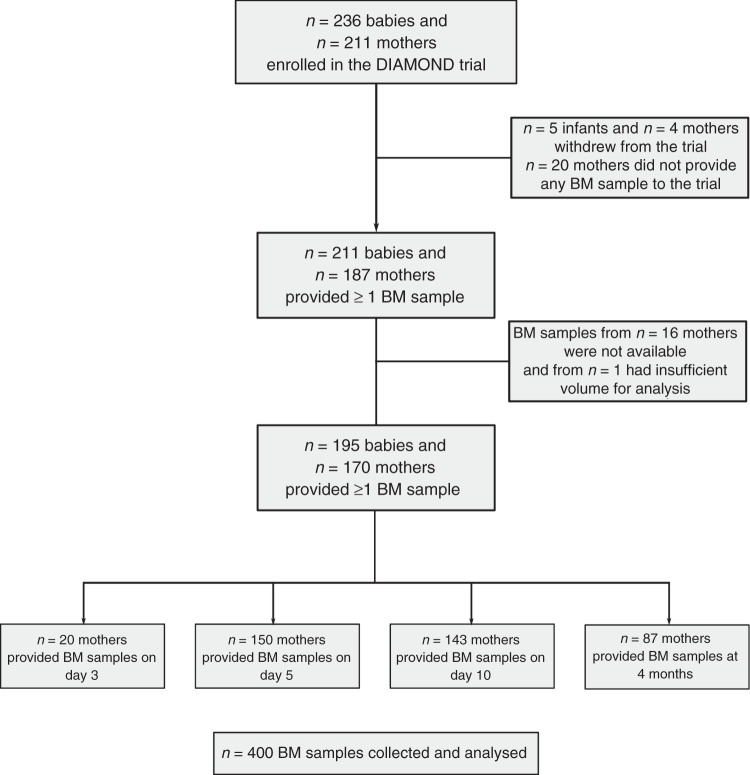
Study flowchart. Numbers do not add up as some mothers provided >1 sample and some mothers gave birth to multiples (twins and triplets). BM breastmilk.

Between July 2017 and August 2019, 400 BM samples were collected from 170 mothers who gave birth to 195 preterm infants recruited into the DIAMOND trial (Fig. 1). Infants were born at a median gestational age of 33 (range 32–35) weeks and most were male (57%), singletons (74%), and recruited at the two Auckland NCUs (76%) (Table 1). Almost 80% of infants were exclusively breastfed at hospital discharge, but only 20% of infants were still being exclusively breastfed at 4 months CA. Most mothers were European or Asian, delivered by cesarean section, received antenatal steroids (57%), and almost 50% were from the two most deprived socioeconomic quintiles (Table 1).

**Table 1:** Participant characteristics.

Recruitment site	
Auckland	80 (41)
Middlemore	68 (35)
North Shore	39 (20)
Waitakere	8 (4)
Maternal characteristics (*n* = 170)	
Maternal age, years	31 (6)
Ethnicity	
European	60 (35)
Māori	23 (14)
Asian	58 (34)
Pasifika	26 (15)
Other	3 (2)
New Zealand Deprivation Index	
Q1 (1,2)	26 (15)
Q2 (3,4)	41 (24)
Q3 (5,6)	22 (13)
Q4 (7,8)	31 (18)
Q5 (9,10)	48 (29)
Education level	
No tertiary education	80 (47)
Complete tertiary education (bachelor, masters, doctoral degree)	90 (53)
Gestational diabetes	34 (20)
Cesarean section	100 (59)
Antenatal steroids course	
None	32 (19)
Incomplete (first dose <24 h from birth)	41 (24)
Complete	97 (57)
Number of samples provided	
1	17 (10)
2	80 (47)
3	69 (40.6)
4	4 (2.4)
Infant characteristics (*n* = 195)	
Boys	112 (57.4)
Gestational age, weeks	33 (32–35)
Birth order	
Singleton	145 (74)
Twins	47 (24)
Triplets	3 (2)
Duration of hospital stay, days	22 (11)
Anthropometric measures	
At birth	
Weight, g	2091 (402)
Length, cm	44.5 (3.0)
Head circumference, cm	31.2 (1.5)
At hospital discharge	
Weight, g	2488 (322)
Length, cm	47.4 (2)
Head circumference, cm	33.0 (1.2)
At follow-up	
Weight, g	6505 (846)
Length, cm	63.6 (2.6)
Head circumference, cm	41.6 (1.3)
Feeding practice	
At hospital discharge (*n* = 194)	
Exclusively breastfed	151 (77.8)
Partially breastfed	37 (19.1)
Exclusively formula fed	6 (3.1)
At follow-up (*n* = 168)	
Exclusively breastfed	36 (21.4)
Partially breastfed	67 (39.9)
Exclusively formula fed	65 (38.7)

Data are presented as *n* (%), mean (standard deviation), or median (range).

### Volatile compounds

We identified forty volatile compounds (Table 2). Most compounds were volatile organic compounds (VOCs, *n* = 9), FAs and FAe (*n* = 9), followed by aldehydes (*n* = 7), terpenoids (*n* = 5), alcohols (*n* = 4), ketones (*n* = 3), and alkyl, furan, and sulfone (*n* = 1 each). Acetone was the most abundant compound, observed in almost 90% of the samples analyzed, followed by octanoic acid methyl ester (56%), *o*-cymene (56%), and decanoic acid methyl ester (52%). The median number of compounds per sample was 4 (range 0–12). Seventeen compounds (VOCs (*n* = 4), terpenoids (*n* = 4), aldehydes (*n* = 4), alcohol (*n* = 2), sulfone (*n* = 1), and FA (*n* = 1)) were detectable in <5 samples (<1%) and were not included in regression models.

**Table 2:** Summary of volatile compounds detected in preterm breastmilk.

Class	Compound	RT	*m*/*z*	*n*	ND	Relative peak area mean (SD)
Alcohol	Amylene hydrate	3.18	59	62	338	673 (345)
	2-Ethyl-1-butanol	6.00	43	15	385	312 (198)
	Ethanol^a^	1.95	31	4	396	44503 (68,482)
	Ethyl acetate^a^	2.55	43	3	397	215 (159)
Aldehyde	Hexanal^b^	6.60	56	126	274	15617 (20,758)
	Butanal, 3-methyl-^b^	3.89	57	25	375	3044 (1880)
	Octanal	13.09	56	15	385	599 (377)
	Pentanal, 3-methyl-^a^	1.81	29	3	397	610 (189)
	Formaldehyde^a^	6.60	56	3	397	304 (82)
	Pentanal^a,b^	3.89	41	3	397	898 (786)
	Cyclohexane carboxaldehyde^a^	12.42	41	2	398	243 (37)
Fatty acid	Octanoic acid^b^	20.74	73	67	333	1766 (2030)
	Pentanoic acid 3-methyl	15.61	60	33	367	1299 (1363)
	Hexanoic acid^b^	15.59	60	23	377	2821 (2244)
	Butanoic acid^a,b^	9.86	60	3	397	2160 (338)
Fatty acid ester	Octanoic acid, methyl ester^b^	16.11	74	224	176	4347 (4290)
	Decanoic acid, methyl ester^b^	21.50	74	209	191	2177 (1847)
	Hexanoic acid, methyl ester^b^	10.03	74	118	282	723 (611)
	Dodecanoic acid, methyl ester^b^	26.34	74	55	345	513 (367)
	Butanoic acid, methyl ester^b^	4.06	74	36	364	555 (269)
Ketone	Acetone^b^	2.02	58	351	49	5189 (7357)
	1-Hepten-3-one	12.48	70	29	371	755 (611)
	Cyclobutanone, 2,2,3-trimethyl-^a^	4.31	41	2	398	127 (102)
Terpenoid	Eucalyptol	12.50	81	38	362	1158 (1941)
	d-Limonene^a^	11.90	67	4	396	67 (57)
	Camphor^a^	17.79	95	3	397	74 (34)
	Fenchone^a^	15.71	81	3	397	304 (82)
	*p*-Cymene^a^	12.50	119	3	397	898 (786)
VOC	*o*-Cymene	12.50	119	224	176	4347 (4290)
	Thrichloromethane	2.67	40	123	277	4577 (13,508)
	Toluene	4.68	91	36	364	2407 (57,412)
	Chloromethane	1.69	50	10	390	88 (56)
	Methane, bromodichloro-	4.11	83	5	395	249 (113)
	Methane, dichloronitro-^a^	2.67	40	4	396	302 (442)
	Ethyl benzene^a^	7.62	91	4	396	406 (112)
	Styrene^a^	8.80	104	3	397	213 (86)
	Benzene^a^	2.84	78	3	397	147 (77)
Alkyl	pentane	1.80	41	26	374	1590 (1806)
Furan	Furan, 2-methoxy-	3.04	83	37	363	59 (62)
Sulfone	Dimethyl sulfone^a^	16.16	94	3	397	27 (9)

*RT* retention time (min), *m/z* mass-to-charge ratio of detected ion, *n* number of samples in which a compound is present, *ND* number of samples in which a compound was not detected, *VOC* volatile organic compounds.

^a^Compounds present in <5 samples and not included in regression models.

^b^Identification confirmed with authentic standards.

### Factors associated with the distribution of volatile compounds in preterm BM

**Fig. 2:**
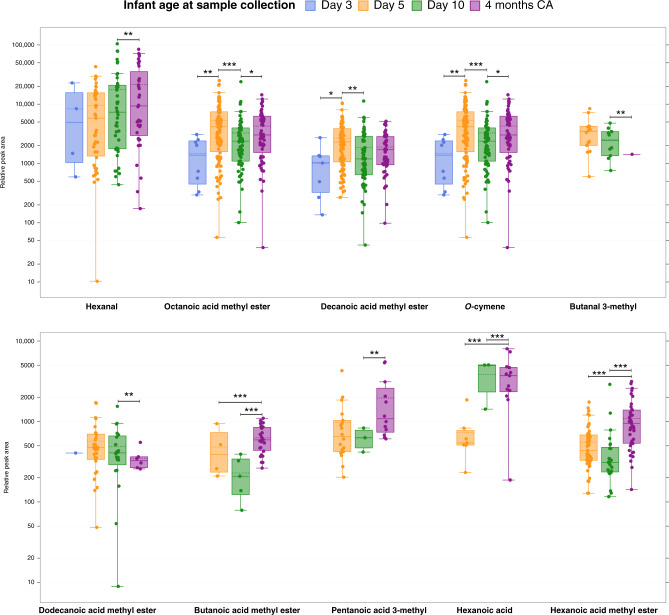
Relative peak area of volatile compounds in preterm breastmilk by infant age at the time of collection. Note the logarithmic scale of the *y*-axis. Boxes represent the median and interquartile range, whiskers represent highest and lowest peak area detected, and dots represent samples. Groups are not shown when the compound was not detected. Significance level is taken as *p* value <0.05, adjusted for comparison (Bonferroni). *p < 0.05; **p < 0.01; ***p < 0.001. CA corrected age.

We explored the association between the distribution of volatile compounds in preterm BM and infant age at sample collection. Twelve compounds differed significantly with infant age (*p* < 0.05) (Supplementary Table 1): seven FA and FAe (Table 3), two aldehydes, one terpenoid, one ketone, and one VOC. Within the colostrum lactation stage, there was a significant increase in relative concentration of octanoic methyl ester (*p* < 0.001), decanoic methyl ester (*p* < 0.001), and *o*-cymene (*p* < 0.001) from postnatal days 3 to 5. Butanal 3-methyl (*p* < 0.01), dodecanoic acid methyl ester (*p* < 0.05), and 1-hepten-3-one (*p* < 0.05) were significantly higher in samples collected on postnatal day 10 compared to mature BM (4 month samples). In contrast, mature BM contained significantly higher relative concentration of butanoic acid methyl ester (*p* < 0.001), hexanoic acid methyl ester (*p* < 0.001), pentanoic acid 3-methyl (*p* < 0.01), hexanoic acid (*p* < 0.001), hexanal (*p* < 0.01), and eucalyptol (*p* < 0.05) compared to samples from colostrum and transitional BM (Fig. 2).

**Table 3:** Association between maternal and infant characteristics and distribution of volatile fatty acids and fatty acid esters in preterm breastmilk.

Characteristics	Pentanoic acid 3-methyl	Hexanoic acid	Octanoic acid	Butanoic acid ME	Hexanoic acid ME	Octanoic acid ME	Decanoic acid ME	Dodecanoic acid ME
Lactation stage	*F*_(3, 329.3)_ = 4.7**	*F*_(3, 329.3)_ = 8.6***	*F*_(3, 303.1)_ = 2.0, *p* = 0.12	*F*_(3, 329.3)_ = 29.6***	*F*_(3, 306.7)_ = 20.5***	*F*_(3, 297.9)_ = 11.8***	*F*_(3, 275.5)_ = 10.2***	*F*_(3, 267.2)_ = 3.6*
Day 3	0 (0)^AB^	0 (0)^A^	0 (0)	0 (0)^A^	0 (0)^A^	592 (1007)^AC^	365 (714)^A^	20 (91)^AB^
Day 5	12 (91)^AB^	16 (912)^A^	423 (1213)	8 (49)^A^	64 (182)^A^	3490 (4768) ^B^	1563 (2058)^B^	106 (283)^A^
Day 10	122 (465)^A^	46 (202)^A^	193 (1132)	13 (92)^A^	217 (402)^A^	1395 (2882)^A^	791 (1525)^A^	68 (213) ^AB^
4 months CA	270 (932)^B^	555 (1558)^B^	314 (672)	194 (319)^B^	514 (740)^B^	2746 (3382)^BC^	1153 (1345) ^AB^	24 (92) ^B^
Ethnicity	*F*_(4, 157.6)_ = 0.42, *p* = 0.8	*F*_(4, 157.7)_ = 1.14, *p* = 0.34	*F*_(4, 160.8)_ = 1.4, *p* = 0.23	*F*_(4, 157.6)_ = 0.54, *p* = 0.71	*F*_(4, 160.4)_ = 1.3, *p* = 0.26	*F*_(4, 160.9)_ = 3.5**	*F*_(4, 162.9)_ = 4.04**	*F*_(4, 163.6)_ = 1.4, *p* = 0.24
European	124 (594)	262 (1142)	405 (1363)	53 (176)	251 (477)	3094 (4300) ^A^	1486 (1907)^A^	93 (254)
Māori	95 (479)	88 (587)	87 (279)	39 (174)	183 (513)	1475 (2580)^AB^	678 (1115)^AB^	32 (103)
Asian	127 (564)	139 (686)	344 (1018)	61 (207)	238 (510)	2619 (3949)^AB^	1237 (1844)^AB^	85 (254)
Pasifika	30 (230)	32 (245)	94 (459)	30 (115)	102 (280)	1281 (3021)^B^	469 (935)^B^	15 (72)
Other	79 (224)	0 (0)	0 (0)	0 (0)	47 (133)	291 (723)^AB^	144 (407)^AB^	0 (0)
GDM	*F*_(1,164)_ = 2.52, *p* = 0.11	*F*_(1,163.9)_ = 0.36, *p* = 0.55	*F*_(1,167.8)_ = 2.54, *p* = 0.11	*F*_(1,163.7)_ = 2.34, *p* = 0.13	*F*_(1,166.8) _= 3.14, *p* = 0.08	*F*_(1,167.9)_ = 4.78*	*F*_(1,169.5)_ = 5.9*	*F*_(1,169.8)_ = 4.45*
No	127 (580)	173 (898)	341 (1158)	56 (191)	235 (487)	2662 (4077)	1263 (1820)	84 (243)
Yes	23 (124)	114 (549)	104 (357)	26 (99)	119 (359)	1463 (2606)	602 (1069)	13 (66)
NZdep	*F*_(4, 153.1)_ = 1.03, *p* = 0.4	*F*_(4,153.1)_ = 4.67**	*F*_(4,157.4)_ = 4.57**	*F*_(4,153.1)_ = 0.74, *p* = 0.57	*F*_(4,156.9)_ = 2.75*	*F*_(4,158.1)_ = 4.81**	*F*_(4, 161.1)_ = 4.33**	*F*_(4, 161.9)_ = 2.61*
Q1	199 (782)	127 (589)^A^	228 (519)^A^	76 (214)	294 (592)^A^	3076 (3672)^AB^	1281 (1552)^AB^	42 (130) ^AB^
Q2	121 (611)	194 (838)^A^	409 (1220)^AB^	59 (206)	270 (527)^A^	2921 (4437)^AB^	1471 (1979)^B^	94 (261) ^AB^
Q3	104 (354)	604 (1880)^B^	871 (2254)^B^	40 (141)	300 (574)^A^	4062 (5853)^A^	1840 (2594)^B^	172 (397) ^†A^
Q4	36 (166)	63 (330)^A^	88 (273)^A^	45 (154)	141 (295) ^†B^	1623 (2326)^B^	816 (1137)^AB^	39 (134) ^AB^
Q5	77 (449.6)	43.7 (387.7)^A^	130.2 (474.7)^A^	34.3 (156)	123.1 (341) ^†B^	1488 (2808)^B^	678 (1243)^A^	47 (158) ^†B^
Antenatal steroids	*F*_(2,153.9)_ = 0.21, *p* = 0.81	*F*_(2,154.5)_ = 0.1, *p* = 0.9	*F*_(2,161.4)_ = 0.65, *p* = 0.52	*F*_(2,153.6)_ = 2.01, *p* = 0.14	*F*_(2,160.1)_ = 2.53, *p* = 0.08	*F*_(2,162.1)_ = 1.71, *p* = 0.18	*F*_(2,165.2)_ = 2.92, *p* = 0.06	*F*_(2,166.1)_ = 0.96, *p* = 0.38
None	100 (443)	148 (990)	149 (579)	50 (197)	128 (378)	1571 (3379)	582 (1025)	25 (95)
Incomplete	142 (624)	208 (835)	360 (1027)	83 (222)	305 (608)	2638 (3972)	1295 (1844)	77 (251)
Complete	94 (505)	147 (798)	314 (1177)	35 (146)	200 (414)	2613 (3941)	1241 (1808)	82 (236)
Tertiary education	*F*_(2,130.4)_ = 0.03, *p* = 0.97	*F*_(2,131.4)_ = 0, *p* = 0.99	*F*_(2,140.2)_ = 0.08, *p* = 0.92	*F*_(2,130.7)_ = 0.02, *p* = 0.98	*F*_(2,139.5)_ = 0, *p* = 0.99	*F*_(2,142.5)_ = 0.36, *p* = 0.7	*F*_(2,150.3)_ = 1.43, *p* = 0.24	*F*_(2,153.2)_ = 0.99, *p* = 0.37
Incomplete	100 (472)	146 (836)	298 (1239)	44 (164)	207 (489)	2263 (4258)	1015 (1761)	59 (216)
Complete	114 (571)	176 (855)	298 (895)	55 (190)	220 (453)	2563 (3543)	1214 (1675)	76 (226)
Delivery mode	*F*_(1,154.7)_ = 1.53, *p* = 0.22	*F*_(1,154.6)_ = 2.09, *p* = 0.15	*F*_(1,161.6)_ = 0.03, *p* = 0.87	*F*_(1,154.5)_ = 2.8, *p* = 0.09	*F*_(1,160.7)_ = 0, *p* = 0.98	*F*_(1,162.3_) = 0.13, *p* = 0.72	*F*_(1,165.4) _= 0.59, *p* = 0.44	*F*_(1,166.3)_ = 0.25, *p* = 0.62
Vaginal	70 (291)	234 (999)	310 (1121)	67 (217)	219 (438)	2410 (3786)	1077 (1667)	65 (216)
C-section	132 (639)	113 (713)	286 (1013)	38 (144)	209 (487)	2451 (3930)	1179 (1760)	74 (227)
Gestational age	*F*_(3,152.3)_ = 0.76, *p* = 0.52	*F*_(3,152.4)_ = 1.27, *p* = 0.29	*F*_(3,159.2)_ = 0.54, *p* = 0.65	*F*_(3,152.3)_ = 0.11, *p* = 0.96	*F*_(3,158.5)_ = 0.1, *p* = 0.96	*F*_(3,160.1)_ = 0.52, *p* = 0.67	*F*_(3,163.3)_ = 0.79, *p* = 0.5	*F*_(3,164.1)_ = 1.26, *p* = 0.29
32	104 (563)	179 (787)	233 (583)	49 (161)	224 (526)	2293 (3576)	1013 (1527)	37 (129)
33	51 (238)	170 (802)	387 (1469)	45 (160)	223 (456)	2749 (4197)	1367 (1911)	101 (258)
34	136 (681)	58 (447)	240 (945)	49 (177)	189 (445)	2125 (3755)	1022 (1662)	64 (209)
35	160 (506)	322 (1399)	338 (954)	63 (232)	225 (441)	2685 (3929)	1141 (1761)	82 (284)
Sex	*F*_(1,151.7)_ = 0.87, *p* = 0.35	*F*_(1,152.1)_ = 0.42, *p* = 0.52	*F*_(1,158.8)_ = 0.03, *p* = 0.85	*F*_(1,151.8)_ = 0.77, *p* = 0.38	*F*_(1,157.8)_ = 0.54, *p* = 0.46	*F*_(1,159.3)_ = 0.78, *p* = 0.38	*F*_(1,162.5)_ = 2.08, *p* = 0.15	*F*_(1,163.4)_ = 1.03, *p* = 0.31
Male	179 (1012)	15 (120)	303 (1233)	37 (155)	29 (261)	2124 (3850)	926 (1603)	54 (198)
Female	135 (657)	28 (144)	288 (926)	57 (190)	8 (118)	2589 (3843)	1250 (1769)	80 (237)

Relative peak area presented as mean (SD).

*ME* methyl ester, *GDM* gestational diabetes mellitus, *NZDep* New Zealand Deprivation Index.

Significance level is taken as *p* value <0.05, adjusted for comparison (Bonferroni).

Different superscript letters demonstrate significant differences in relative peak area of that compound within the row category.

*p  <  0.05; **p  <  0.01; ***p  <  0.001.

^†^Adjusted *p* > 0.05 (Bonferroni).

**Fig. 3:**
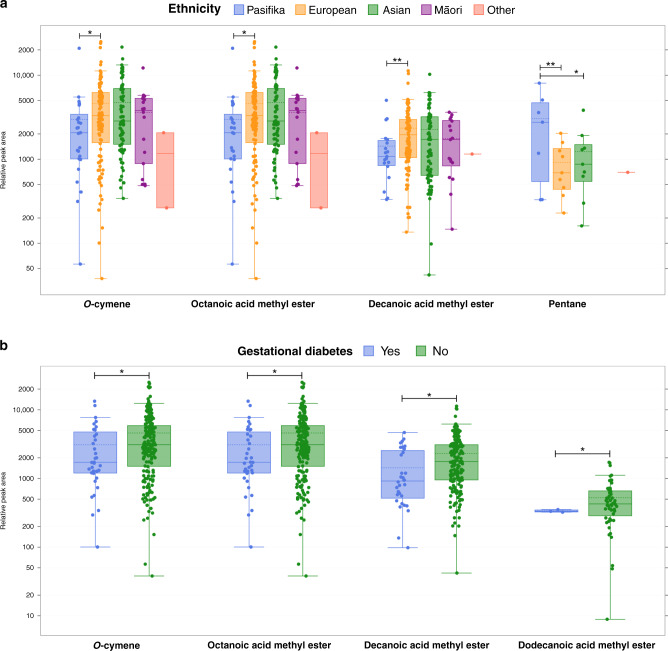
Relative concentration of volatile compounds in preterm breastmilk by maternal ethnicity and gestational diabetes. (**a**) Maternal ethnicity; (**b**) gestational diabetes. Note the logarithmic scale of the *y*-axis. Boxes represent the median and interquartile range, whiskers represent highest and lowest peak area detected, and dots represent samples. Groups not shown when compound was not detected. Significance level is taken as *p* value <0.05, adjusted for comparison (Bonferroni). *p < 0.05; **p < 0.01.

BM samples from European mothers had a higher relative concentration of *o*-cymene, octanoic, and decanoic acid methyl esters compared to BM samples from Pasifika (*p* < 0.01) (Table 3 and Supplementary Table 2). Pentane was significantly higher in BM from Pasifika mothers compared to Māori and Asian mothers (*p* < 0.01) (Fig. 3a).

The relative concentrations of three FAe (octanoic, decanoic, and dodecanoic acid methyl esters) (Table 3) and one VOC (*o*-cymene) were significantly lower in BM from mothers with GDM compared to mothers without GDM (all *p* < 0.05, Fig. 3b and Supplementary Table 3).

**Fig. 4:**
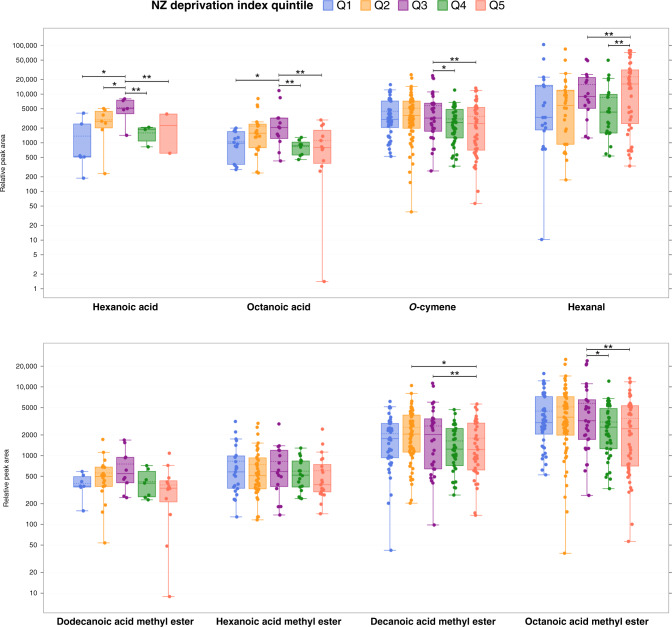
Relative concentration of volatile compounds in preterm breastmilk by New Zealand Deprivation Index quintiles (1, low; 5, high). Note the logarithmic scale of the *y*-axis. Boxes represent the median and interquartile range, whiskers represent highest and lowest peak area detected, and dots represent samples. Groups are not shown when the compound was not detected. Significance level is taken as *p* value <0.05, adjusted for comparison (Bonferroni). *p < 0.05; **p < 0.01. Q quintile.

Eight compounds differed across NZdep quintiles (Supplementary Table 4). These were: hexanal (*p* < 0.001); the methyl esters of hexanoic acid (*p* < 0.05), octanoic acid (*p* < 0.01), decanoic acid (*p* < 0.01), and dodecanoic acid (*p* < 0.05); hexanoic acid (*p* < 0.01), octanoic acid (*p* < 0.01), and *o*-cymene (*p* < 0.01) (Table 3). BM samples from mothers of the most deprived quintile presented significantly lower relative concentration of *o*-cymene, octanoic, and decanoic methyl esters compared to BM samples from mothers belonging to the third quintile of deprivation (*p* < 0.05). In contrast, hexanal was significantly higher in BM of mothers from the most deprived quintile (Q5) in relation to mothers from least deprived areas (Fig. 4).

There were no associations between the distribution of volatile compounds in preterm BM and other maternal characteristics such as delivery mode, antenatal steroids, education level, and maternal age. There were no associations between the distribution of volatile compounds in preterm BM and infant sex, birth weight, or length of gestation.

## Discussion

Using an untargeted approach, we detected forty volatile compounds in preterm BM, the majority of which were FAs and FAe and VOCs, but aldehydes, ketones, and terpenoids were also present. Semi-quantitative analysis demonstrated that the profile of volatile compounds in preterm BM is influenced by maternal, but not infant, characteristics. Stage of lactation, and even day of collection within the colostrum stage, maternal ethnicity, gestational diabetes, and social deprivation index were all significantly associated with the profile of volatile FA and FAe.

Previous studies suggested that the profile of volatile compounds in BM results from degradation of milk lipids (yielding ketones, aldehydes, alcohols, free FAs, and FAe) and from maternal diet (mostly terpenoids).^30,38–41^ BM from ten mothers of FT infants was found to contain higher amounts of terpenes, aldehydes, and alcohols than infant formulas,^38^ although, in contrast to our findings, that study only detected SCFA in the BM. However, samples analyzed in this study were all from mature BM (>5 weeks postnatal) provided by mothers of FT infants, which may explain the different results.^38^

To the best of our knowledge, volatile compounds in preterm BM have been reported only twice previously and in small numbers. A study of four BM samples analyzed by GC/olfactometry reported five volatile compounds (two ketones, two alcohols, and one aldehyde).^40^ We previously reported that volatile compounds in 15 samples of preterm BM differed from those in infant formulas largely due to the presence of specific FA in BM that was absent from formulas.^30^ Therefore, our findings are largely in agreement with previous reports and expand on the current knowledge of volatile compounds in BM by demonstrating that a variety of compounds of different chemical classes can be found in preterm BM, including MCFA and their esters, terpenoids, and VOCs.

The lipid fraction of human milk, in the form of milk fat globules (MFGs),^24^ is the most variable macronutrient in BM composition, responsible for provision ~50% of total energy for the growing infant.^42,43^ The MFG is a complex structure formed by a triacylglycerol (TAG) core, protected by a highly specialized membrane composed of phospholipids, sphingolipids, cholesterol, bioactive proteins, and long-chain polyunsaturated FAs (LCPUFAs) that is unique to the human species.^24,42,44^

The FA composition of the TAG core is ~42% saturated FAs (mostly MCFAs), 42% monounsaturated FAs, and 16% polyunsaturated FAs (PUFAs).^42^ Long-chain saturated FAs and PUFAs are mainly sourced from the maternal circulation and body stores; saturated MCFAs are synthesized in the mammary epithelial cells and incorporated into TAGs for secretion into MFG.^42,43^ The de novo synthesis of MCFA from glucose in the mammary epithelial cells is influenced by substrate availability (from circulation or body stores) and the action of hormones such as prolactin, growth hormone, and insulin.^42,43^ Thus, the composition of TAG can be modified by maternal factors that influence those hormones or the supply of FA and glucose precursors, such as diet and supplementation,^22,45,46^ length of gestation,^27^ lactation stage,^27,42^ and socioeconomic status.^47^

Previous studies report an increase in the content of FA in BM as the lactation stage progresses,^27,42^ consistent with our findings. Mothers of preterm infants have been reported to secrete BM with higher amounts of MCFA throughout lactation compared to BM from mothers of full-term infants.^27^ We observed greater relative concentrations of octanoic and decanoic methyl esters in colostrum, with a significant increase from postnatal days 3 to 5. This may be advantageous to preterm infants as MCFA can be more easily converted into energy^42^ and may exhibit antimicrobial activity.^48^ Unlike long-chain saturated FAs that are packed into chylomicrons and transferred to the liver via the lymphatic system for later utilization, MCFAs are readily solubilized in the enterocyte, absorbed bound to albumin, and released into the portal system. Upon reaching the liver, they are either immediately oxidized (generating energy) or transferred to fat stores.^49^ This improved energy bioavailability in colostrum and possible protection against pathogens may be advantageous for transition to the postnatal environment in immature infants with high metabolic demand and limited endogenous lipase activity.^50^

Alternatively, the presence of volatile MCFA in preterm BM may relate to immaturity of the mammary gland and inefficient milk secretion following a shorter gestation period, resulting in temporary increased secretion of TAGs with higher content of MCFA, which may resolve as lactation progresses.^42^ It is notable that free FA can bind to G-protein-coupled taste and smell receptors located throughout the gastrointestinal tract, influencing the secretion of hormones associated with digestion and metabolism.^51,52^ Nevertheless, the reason for higher MCFA in colostrum compared to transitional BM is not yet clear.

We found that maternal factors were significantly associated with the distribution of MCFA volatile compounds in preterm BM. Recent meta-analysis of FA composition in BM from various countries found that saturated FA in BM, including MCFA, can vary between 39 and 50% of total milk fats.^23^ One small study of 78 multiethnic lactating mothers conducted in New Zealand found that, although the composition of BM did not differ between mothers of different ethnicities, PUFA content in BM from Asian mothers was significantly higher than in BM from European and Māori/Pasifika mothers^53^ even though maternal diets were similar in terms of energy and macronutrients. However, consumption of specific foods differed, with the intake of chicken and legumes significantly higher in Asian mothers and consumption of dairy product significantly higher in European mothers.^53^

It is known that the carbohydrate-to-fat ratio of the maternal diet modulates the profile of FA in BM.^22,43^ Therefore, the ethnic differences in the profile of MCFA observed in our study may relate to maternal diet and habits. However, the lack of maternal dietary information to further assess this is a limitation of our study.

Maternal socioeconomic deprivation also was associated with the distribution of FA and FAe volatile compounds of preterm BM. An Iraqi study reported that mothers of low socioeconomic status produced mature BM with a significantly lower content of TAGs, total cholesterol, phospholipids, and the saturated FAs decanoic and dodecanoic acid (smaller FA were not analyzed) compared to mothers with a higher socioeconomic status.^47^ Our results are consistent with these findings, as we also found significantly lower relative concentrations of FA and FAe in BM of the most socially deprived mothers.

Interestingly, the relative concentration of hexanal was significantly higher in mature BM and in samples collected from most socially deprived mothers (Q5). Hexanal is a secondary product originating from oxidation of omega-6 FAs and its presence in BM and infant formula can indicate lipid degradation.^54^ It has been demonstrated that the formation of hexanal from lipid peroxidation in BM is inversely correlated with the concentration of vitamins E and C.^54^ Vitamins are powerful antioxidants and, therefore, their antiantioxidant capacity may prevent lipid oxidation of BM.^54^ Thus, considering the influence of maternal diet on the distribution of volatile compounds in BM,^30,38,40^ it seems possible that the elevated concentration of hexanal in BM of the mother from most deprived socioeconomic quintile could be related to reduced antioxidant components in their BM. Further research is required to confirm this association.

We found that BM from mothers with GDM had lower relative concentrations of methyl esters compared to mothers without GDM. Several studies have suggested that GDM influences the composition of BM, but the evidence is conflicting.^55^ While higher concentrations of omega-6 LCPUFAs in colostrum of mothers with GDM^56^ has been reported, others found lower concentrations of organic acids and unsaturated FA in BM of mothers with GDM compared to mothers without GDM;^57^ others reported no differences in colostrum and transitional BM macronutrient composition between mothers with and without GDM, except for a slightly higher fat and energy content in BM of mothers without GDM.^58^ These studies raise the possibility that inappropriate glucose homeostasis may influence FA metabolism leading to an altered profile of FA in BM.^55,56,58^ Obesity and insulin dependency are known risk factors for delayed onset of Lactogenesis II.^59–61^ Hence, it is possible that varying levels of circulating glucose and insulin in mothers with GDM disrupt de novo synthesis of MCFA in the mammary epithelial cells or delayed onset of Lactogenesis II, which in turn would lead to a TAG deficient in MCFA, consistent with our findings.

In addition to FA and FAe, we have also identified several compounds classified as VOCs. The presence of VOCs in BM, such as benzene, toluene, styrene, bromochloronitromethane, and others, has been correlated with indoor exposure to pyrogenic and petrogenic air pollutants.^29,62^ The main entry routes into the human body are inhalation, ingestion, and dermal absorption.^29,62,63^ Alternatively, VOCs like *o*-cymene have been detected in kitchen cleaning agents and air fresheners.^64^ We found that the relative concentration of *o*-cymene was more abundant in BM from European mothers than from Pasifika mothers, and significantly less abundant in BM from mothers with GDM, those from the most socially deprived quintiles, and in transitional BM compared to colostrum and mature BM samples. Similarly, toluene was also detected in our study and might originate from exposure to air pollutants,^65^ but may also originate from degradation of dietary β-carotene.^66^ Although previous studies have detected only low levels of VOCs in BM, the lipophilic nature of these compounds suggests that bioaccumulation in fat tissue deposits may occur,^29,62–64^ and these could, in turn, be mobilized during BM fat synthesis. Nevertheless, the limited information available from maternal diet and exposure to environmental contaminants prevents us from determining the origin of these compounds in preterm BM.

We have demonstrated that the odor of preterm BM comprises a mixture of FA and their esters and ketones and, to a lesser extent, aldehydes and terpenoids. FAe have low perception threshold (at parts per billion level), meaning that even when present in low concentrations these compounds can contribute to the aroma of a food matrix.^67,68^ Similarly, ketones also have low odor perception thresholds.^69^ The odor characteristics of both of these compounds are described as fruity, malty, almond, and fatty.^67,69^ FAs also contribute to the overall aroma of food; however, these compounds have higher odor perception thresholds (at parts per million level), compared to esters and ketones,^69^ meaning that higher concentrations are required for odor perception.

The concentration of specific volatile compounds and their interaction with other compounds present in the food matrix is important for the overall aroma of food.^30^ For example, free FAs present in low concentrations in the food matrix may contribute positively to the aroma of food; however, when present in high concentrations they may confer off-notes such as fishiness and rancidness,^68^ influencing overall palatability of food. Similarly, FAe can contribute positively to the aroma of food in low concentrations, but their contribution to overall flavor perception can be negatively affected by interaction with other FAe present in the food matrix, leading to development of unpleasant smells.^67^ Accordingly, our results indicate that the odor of preterm BM is largely influenced by the concentration of FA and FAe, but ketones, aldehydes, and terpenoids might also contribute to the overall smell of BM.

### Strengths and limitations

Some limitations of our study should be acknowledged. First, no information on maternal diet or supplementation, anthropometry, smoking, and exposure to environmental toxins was collected, preventing us from drawing further conclusions about the source of some volatile compounds identified in preterm BM. Second, as samples were collected during the morning, it is possible that this resulted in our measuring lower fat content as BM produced towards the end of the day contains higher fat concentrations.^19^ The number of day 3 samples is low as a collection so soon after preterm birth was challenging. Nevertheless, our findings of significant changes in the volatile profile between days 3 and 5 suggest that further investigation of changes in BM composition during the colostrum stage is warranted. Samples were analyzed up to 24 months after collection and frozen storage of BM for long periods may increase volatile products of lipid degradation, such as aldehydes and FA.^70^ We did not find any association between the distribution of volatile compounds in preterm BM and time elapsed between sample collection and analysis (data not shown), perhaps because samples were stored at very low temperature (−80 °C).^71,72^ Thus, we are confident that lipid degradation has not biased the final results.

Our study also has several strengths. Sample collection followed a strict protocol to minimize technical variations.^34^ For the first time, volatile compounds have been analyzed in a large number of samples from colostrum, transitional and mature BM, enabling assessment of longitudinal changes in the profile of volatile compounds. Further, an untargeted approach is a useful tool for the discovery of volatile compounds in preterm BM^73^ and can serve as a screening method for future quantification studies. In addition, by extracting the volatile compounds at a temperature similar to feeding (37 °C), we were able to assess which compounds are likely to be perceived by infants during feeding, expanding current knowledge on sensory properties of preterm BM.

### Future directions

Considering that early life sensory exposure may impact later life feeding behavior and preferences,^8^ future studies should aim to determine the contribution of maternal diet and exposure to environmental contaminants to the profile of volatile compounds in BM. Quantification of environmental contaminants and determination of their origin in preterm BM from different populations may be warranted, as exposure to persistent organic pollutants may influence the growth of preterm infants.^28^ In addition, the association between various maternal characteristics and volatile compounds in BM and whether differences in volatile compounds in BM affect feeding behavior or metabolism in preterm babies are yet to be determined.

## Conclusion

Sensory-active volatile FAs and their esters are the major contributors to the smell of preterm BM. Our findings demonstrate that the profile of volatile compounds in BM is influenced by maternal but not by infant characteristics. Lactation stage, ethnicity, gestational diabetes, and socioeconomic status are associated with the distribution of volatile FA and FAe in preterm BM. Future studies are required to investigate the origin of volatile compounds in preterm BM and their implications for the nutrition of preterm infants.

## Supplementary information

Supplementary Checklist

Supplementary Information
